# Use of information sources regarding medicine side effects among the general population: a cross-sectional survey

**DOI:** 10.1017/S1463423619000574

**Published:** 2019-12-10

**Authors:** Bernadine O’Donovan, Ruth M. Rodgers, Anthony R. Cox, Janet Krska

**Affiliations:** 1 National Cancer Registry Ireland, Kinsale Road, Cork, Ireland; 2 Medway School of Pharmacy, Universities of Kent and Greenwich at Medway, Anson Building, Central Avenue, Chatham Maritime, Kent, UK; 3 School of Pharmacy, College of Medical and Dental Sciences, University of Birmingham, Edgbaston, Birmingham, UK

**Keywords:** adverse drug reaction, medicine information, patient experiences

## Abstract

**Aim:**

To determine the use and perceived value of different information sources that patients may use to support identification of medicine side effects; to explore associations between coping styles and use of information sources.

**Background:**

Side effects from medicines can have considerable negative impact on peoples’ daily lives. As a result of an ageing UK population and attendant multi-morbidity, an increasing number of medicines are being prescribed for patients, leading to increased risk of unintended side effects.

**Methods:**

A cross-sectional survey of patients who use medicine, recruited from community pharmacies. The survey sought views on attributes of various information sources, their predicted and actual use, incorporating a shortened Side Effects Coping Questionnaire (SECope) scale and the abbreviated Miller Behavioural Style Scale (MBSS).

**Findings:**

Of 935 questionnaires distributed, 230 (25.0%) were returned, 61.3% from females; 44.7% were retired and 84.6% used at least one medicine regularly. 69.6% had experienced a side effect, resulting in 57.5% of these stopping the medicine. Patient information leaflets (PILs) and GPs were both predicted and actually most widely used sources, despite GPs being judged as relatively less accessible and PILs less trustworthy, particularly by regular medicine users. Pharmacists, considered both easy to access and trustworthy, were used by few in practice, while the internet was considered easy to access, but less trustworthy and was also little used. SECope sub-scales for non-adherence and information seeking showed positive associations with stopping a medicine and seeking information from a health professional. More high monitors than low monitors stopped a medicine themselves, but there were no differences in use of information sources. Information seeking following a side effect is a common strategy, potentially predicted by the SECope, but not the MBSS. Limited GP accessibility could contribute to high internet use. Further research could determine how the trustworthiness of PILs can be improved.

## Introduction

Coping styles are an essential component of health information-seeking behaviours. Coping styles are a mixture of personal characteristics and attributes, such as self-efficacy and locus of control, which form an essential component of health information-seeking behaviours. People have distinctive coping styles/attentional styles when faced with threatening situations and can engage in a wide range of responses to the stress (Miller, 1989). These coping responses/behaviours can be classified as active cognitive strategies, such as information seeking and self management, and passive coping strategies, such as catastrophising and wishful thinking (Sahler and Carr, 2009).

Those with an active coping style or ‘monitors’ tend to engage in information-seeking behaviours in contrast to those with a non-active coping style or ‘blunters’, who use distraction and reinterpretation techniques to lessen the threat (Miller, 1989). Adverse drug reactions (ADRs) are one situation where coping style could affect behaviour. ADRs describe all types of undesired or unpredicted medicine-induced effects. These are sometimes referred to as side effects and can include beneficial as well as harmful therapeutic outcomes (DeWitt and Sorofman, 1999; Edwards and Aronson, 2000). Active information seekers may gather a large amount of information about their medical conditions and potentially also about side effects of medicines, as information can help to alleviate their anxiety and stress in relation to their health. Based on this theory, it can be hypothesised that patients with other coping styles may not actively seek information about side effects.

Much work has shown that many patients actively seek information about health issues, including medicines, and use a variety of sources (Hughes *et al*., 2002; Nähri and Helakorpi, 2007; Munksgaard *et al*., 2011; Clarke *et al*., 2016). Six general principles of information-seeking behaviour have been identified (Harris and Dewdney, 1994) as follows:

1. Information needs arise from the person’s situation.
2. The decision to seek/not to seek information is influenced by numerous factors.
3. People tend to seek the most accessible information.
4. People tend to first seek information from interpersonal sources, especially from people like themselves.
5. Information seekers expect emotional support.
6. People follow habitual patterns in seeking information.

Relevant information about medicines can lead to positive adherence and treatment outcomes (Nähri and Helakorpi, 2007).

A study in Finland found that patient information leaflets (PILs), legally required to be supplied with all licensed medicines in Europe, were the most commonly used information sources (74%), followed by doctors (68%) and pharmacists (60%), while 40% used television, 40% print media, 24% family/friends, 22% medicine books and 20% the Internet particularly among younger people (Nähri and Helakorpi, 2007). Research has also been conducted into PIL use in England – a study of pharmacy customers in England found that the majority of first-time users of prescription medicine used PILs (71%), while 87% used PILs at some point. While the side effect section in PILs was the most commonly read section, many frequent medicine users (60%) never/rarely used PILs after their first use (Raynor *et al.*, 2007). A later study of hospital patients in England found use of PILs differed depending on patients’ experiences of ADRs. In general, 19% never read the PIL and over half (56%) never sought further information about possible side effects from their medicines. Over half of the patients who experienced a suspected ADR had read the PIL (54%). However, 36% of these patients only did so after the ADR had occurred (Krska and Morecroft, 2013). Sources such as PILs, the internet or health professionals are used by many patients to confirm their suspicions about ADRs (Krska and Morecroft, 2013). However, the majority of patients initially employ temporal associations to link symptoms to a medicine (Krska *et al.*, 2011; Chaipichit *et al.*, 2014).

It is clear that the majority of people from most countries prefer to get information in person from a health professional (Munksgaard *et al.*, 2011; Clarke *et al.*, 2016), although they may in practice use multiple sources of information (Nähri and Helakorpi, 2007; Clarke *et al.*, 2016), which could lead to information overload (Clarke *et al.*, 2016), contradictory information and even negatively impact on adherence (Carpenter *et al.*, 2010). The trustworthiness of the information source could be a key issue in patient preferences. In general, PILs and health professionals such as doctors and pharmacists are viewed across all age groups as trustworthy sources of information (Nähri and Helakorpi, 2007; Clarke *et al.*, 2016). For the internet, trustworthiness of a particular website may be determined by its professional appearance (Nicolson *et al.*, 2011), but as a source of information it may be preferred because of its accessibility (Clarke *et al*., 2016). Media sources are regarded as less trustworthy, although potentially useful for informing people about the risks and benefits of medicines (Moynihan *et al.*, 2000), and some studies do show relatively high use of the media as information sources (Nähri and Helakorpi, 2007; Clarke *et al.*, 2016).

To our knowledge, no research has explored the use of information sources regarding side effects of medicines in relation to coping styles. This study therefore aimed to determine the use and perceived value of different information sources to support side effect identification. It also explored associations between coping styles and use of information sources with three sources of information – direct questions and two embedded scales.

## Method

### Inclusion criteria

Adults aged 18 or over, resident in the UK, who had used prescription or purchased medicines in the past six months and were able to complete questionnaire, which was only available in English.

### Ethics

A favourable ethical opinion was obtained from the Proportionate Review Sub-committee of the NRES Committee North East - Newcastle & North Tyneside 1 (REC ref: 14/NE/1053).

### Questionnaire development

The questionnaire was developed iteratively within the research team to seek views on the accessibility, trustworthiness, relevance and ease of understanding of a range of information sources about medicine side effects, their predicted use and actual use on experiencing a side effect. It also sought details of any side effect experience occurring within the previous six months, including symptoms, severity, suspected medicine and consequences. Questions were based where possible on previous research instruments, and a mix of open-ended and closed questions were used (McLernon *et al.*, 2011; Krska and Morecroft, 2013). Free-text questions were included to gather information on the impact of the side effect(s) on their daily lives and how they concluded that the medicine had caused the side effect(s). Two validated instruments were incorporated into the questionnaire: the abbreviated Side Effects Coping Questionnaire (SECope; de Smedt *et al.*, 2011), to determine potential coping strategies, and the abbreviated Miller Behavioural Style Scale (MBSS; Steptoe, 1989), to determine coping styles. Demographic characteristics were also included. The Medway School of Pharmacy public engagement group – Public Involvement in Pharmacy Studies Group – also assessed and provided feedback on the questionnaire.

### Piloting

Initial piloting involved providing the questionnaire by hand to people known personally to the research team, known to have experienced a side effect, while the proposed distribution method was also piloted separately in a single community pharmacy. Both groups received an envelope containing the questionnaire, information sheet, feedback form and prepaid envelope for return and were asked to assess the questionnaire in terms of clarity, ease of completion, face validity and overall functionality. Several modifications were made after this pilot to clarify the layout and instructions and to shorten the SECope questionnaire. This was followed by a second pilot, involving a novel group of participants, either known to the research team or recruited through snowballing, all of whom had experienced a side effect. These participants received the revised questionnaire and feedback form by post with a prepaid envelope for return and were also asked to assess the questionnaire for clarity, ease of completion and functionality. No further modifications were required after this second pilot study.

### Main survey distribution

Superintendents of small-to-medium chain pharmacies in areas of [Anonymised for review], known to the research team, were contacted, and if willing to allow questionnaire distribution, invitation letters were sent to pharmacists working in pharmacies owned by these chains. In those pharmacies which agreed, the questionnaires were distributed by hand, to adult customers after initial screening to ensure they had used at least one medicine in the previous six months. Each customer waiting for a prescription to be filled was approached by the researcher. The researcher outlined the study and invited people to participate. If the individual indicated their willingness to participate, they were asked questions to determine if they satisfied the inclusion criteria for the study. Potential participants received an information sheet, questionnaire and prepaid envelope for return. They were asked to complete the questionnaire at their leisure and return the completed questionnaire in the prepaid envelope.

### Data analysis

Responses from returned questionnaires were entered into SPSS version 23. Demographic variables and responses to closed questions were analysed using simple descriptive statistics. Responses to open-ended questions were entered into the data management program NVivo. Content analysis was used to code these and identify points of commonality. Chi-squared tests for categorical data and analysis of variance or *t*-tests for continuous data were used to assess differences between sub-groups and Spearman correlation coefficient calculated to assess relationships between continuous variables.

### Analysis of SECope

The SECope scale as modified during piloting consisted of 10 statements describing actions which could be taken in response to experiencing a suspected side effect, together with a 5-point scale indicating likely frequency of taking this action, ranging from never to always. Respondents were required to consider each action and indicate the likelihood of them taking it. Numerical values were assigned to the responses and summed to obtain overall scores, with one item being reverse scored. Factor analysis was carried out on this 10-item scale, using principal component analysis (PCA), to ensure that it still covered the constructs contained within the original scale. Internal reliability was assessed using Cronbach’s alpha, accepting a value of 0.7 as indicating consistency. The PCA is reported in the Results section.

### Analysis of MBSS

The abbreviated MBSS consists of two hypothetical scenarios – going to the dentist and threat of job loss – both with eight possible choices, four describing monitoring coping styles, four blunting coping styles, each with four response options ranging from complete disagreement (never) to complete agreement (always). Respondents were required to visualise themselves in the scenarios and respond to each option, indicating how they believe they would behave. Three scores were generated: a total monitoring score, a total blunting score and a summary MBSS score (calculated by subtracting blunting from monitoring scores). Subjects with a score above the median were categorised as high monitors and those with scores below the median as low monitors (or blunters).

## Results

### Response rates and demographic details

A total of 48 questionnaires were distributed in the first pilot, from which 28 questionnaires were returned (58%), and 20 distributed in the second pilot, with 15 (75%) returned. These data were not included in analysis. In the main survey, 935 questionnaires were distributed (855 in [Anonymised for review] and 80 in the [Anonymised for review]), from which 230 (25.0%) were returned.

Over half of the respondents were female (141; 61.3%), 73 (32.0%) were aged below 50, 99 (43.4%) were aged 51–70 and 56 (24.6%) were above 70 years; 164 (71.9%) were of White ethnicity (Table 1). Educational level ranged from 61 (26.5%), who left school at 16, to 72 (31.7%) who were educated to University level. A high proportion of respondents (102; 44.7%) were retired. Most (193; 84.6%) used at least one medicine regularly.

**Table 1.** Demographic details of survey respondents (*n* = 230)

Characteristic		Number (%)
Gender	Female	141 (61.3%)
Age group	<40	44 (19.3%)
	41–50	29 (12.7%)
	51–60	49 (21.5%)
	61–70	50 (21.9%)
	71–80	44 (19.3%)
	>80	12 (5.3%)
Educational level	Left school at 16 or younger	61 (26.5%)
	Left school at 17 or 18	36 (15.7%)
	Further education	60 (26.2%)
	Higher education	72 (31.4%)
Ethnicity	White	164 (72.2%)
	Asian	36 (15.9%)
	Black	11 (4.8%)
	Chinese	6 (2.6%)
	Mixed/other	10 (4.4%)
Employment	Full time	64 (28.1%)
	Part time	28 (12.3%)
	Retired	102 (44.7%)
	Student	10 (5.3%)
	Unemployed	12 (4.4%)
Number of regular medicines	One	46 (20.2%)
	2–4	82 (36.0%)
	5–8	53 (23.2%)
	>8	12 (5.3%)
	None	35 (15.4%)

### Information sources: predicted use versus actual use

Respondents’ actual use of information sources to confirm their side effects differed from their predicted use of these sources.

### Predicted use of information sources

**Figure 1.**
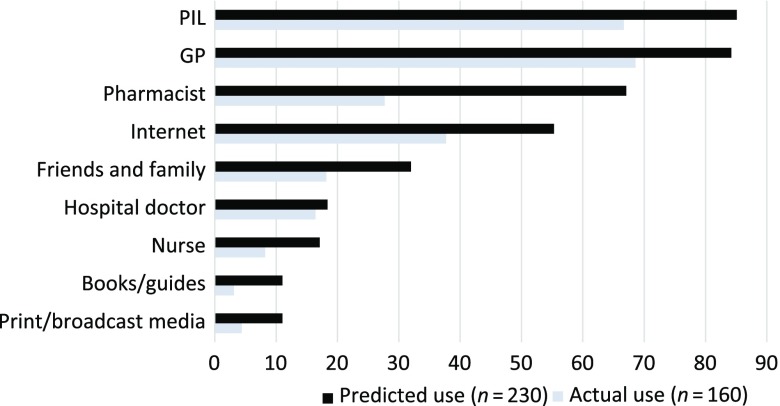
Predicted and actual use of information sources about ADRs

The information sources selected most frequently if they needed to find out about a side effect from a medicine were: PILs (194; 85.1%), GPs (192; 84.2%), pharmacists (153; 67.1%) and the internet (123; 55.3%) (Figure 1).

### Actual use of information sources

There were 160 respondents (69.6%) who had experienced a side effect from a medicine, 71 (30.9%) once and 89 (38.7%) more than once, although for 78 (49.1%) this occurred more than six months previously. A paired comparison analysis indicated that the two most common sources these respondents actually used when seeking information about their side effect experience were similar to the predicted use – GPs and PILs. This group used GPs slightly more frequently than PILs, 68.8% and 66.8%, respectively. However, the proportion who used the internet was much less than that predicted, falling from 92 (58.6%) to 59 (37.6%). Pharmacists were used by only 27.4% (43), in contrast to the 65% (102) who said they would do so (Table 2).

**Table 2.** Predicted use versus actual use of information sources (*n* = 160)

Source	Said would use	Did use in practice	Significance level^a^
GPs (*n* = 157)	134 (85.4%)	108 (68.8%)	<0.001
Hospital doctors (*n* = 157)	31 (19.7%)	16 (10.2%)	0.486
Pharmacists (*n* = 157)	102 (65.0%)	43 (27.4%)	<0.001
Nurses (*n* = 157)	26 (16.6%)	13 (8.3%)	0.021
PIL (*n* = 157)	135 (86.0%)	105 (66.8%)	<0.001
Print and broadcast media (*n* = 157)	12 (7.6%)	7 (4.5%)	0.302
Medicine books (*n* = 157)	14 (8.5%)	5 (3.2%)	0.022
Relatives and friends (*n* = 157)	41 (26.1%)	29 (18.5%)	0.067
Internet (*n* = 157)	92 (58.6%)	59 (37.6%)	<0.001

a McNemar’s test (non-parametric test for related samples).

### Perceived value of information sources

Analysis was conducted on respondents’ assessments of information sources for ease of access, ease of understanding, trustworthiness and relevance.

PILs were considered easy to access by most respondents (180; 78.3%), but the proportion who considered them trustworthy was lower (135; 59.0%) and only 124 (54.1%) judged PILs easy to understand (Table 3). In contrast, most people (181; 79.0%) considered GPs trustworthy information sources, but fewer thought them easy to access (95; 41.5%). Pharmacists were judged both easy to access (175; 76.4%) and trustworthy (166; 72.5%). The internet was viewed as easy to access (146; 64.8%), but only 34 (14.8%) viewed it as trustworthy.

**Table 3.** Attributes of information sources (*n* = 230)

		Proportion indicating agreement with attribute (%)			
Information source		Ease of access	Ease of understanding	Personal relevance	Trustworthiness
Health professionals	GP	41.5	65.5	65.1	79
	Pharmacist	76.4	65.9	52.4	72.5
	Hospital doctors	9.2	26.2	27.1	52.4
	Nurses	30.1	47.2	30.6	44.1
Printed sources	PIL	78.3	54.1	48	59
	Print/broadcast media	30.6	17.5	7.4	8.3
	Books/guides	19.7	13.1	13.1	28.8
Informal sources	Internet	63.8	35.8	28.4	14.8
	Friends/family	50.7	40.2	28.8	28.4

Age group only affected potential use of the internet, with older age groups less likely to use it and considering it less accessible and understandable than younger age groups. Trust in information sources did not differ by age, gender or education. However, fewer respondents using one or more medicines considered PILs trustworthy (106/192; 55.2%) compared to respondents not using regular medicines (25/35; 71.4%) (*P* = 0.006).

### Number and types of information sources used to confirm side effect

Most respondents indicated using more than one source to confirm their assessment of the experience as an ADR. A fifth (31; 19.5%) used only one source, but 50 (31.4%) used two sources, 53 (33.3%) three sources and 25 (15.7%) more than three.

The types of sources used to confirm side effects varied across respondents – of the 31 using one information source, 19 accessed a healthcare professional, 8 used the PIL and 4 the internet. All but six of those using two or more sources accessed a health professional, thus overall 141/160 (88.1%) accessed a health professional. Internet use increased with increasing number of sources.

### Types of information sources and text box responses

This pattern of information used to confirm side effects was further supported by analysis of the text box responses. There were 43 (29%) who described contact with a health professional: *‘Spoke to my GP and he told me it is a common side effect’* (female, 71–80 years, retired, 5–8 medicines). PILs were used by 38 (26%) to confirm their suspicions: *‘I looked in the leaflet of the medication and one of the side effects was stomach upset’* (male, 61–70 years, retired, 1 medicine), but only 5 (3%) mentioned other people: *‘Talking to others on the same tablet – same side effect’* (male, >80 years, retired, >8 medicines). However, although a large proportion indicated they had used the internet to search for information, only two (1%) described this in their own words, both of whom also described using other sources: *‘It was clearly detailed in the information leaflet. Also confirmed on various internet sites’* (male, <40 years, working full time, 1 medicine).

Additional ways of identifying a suspect medicine described in free-text comments illustrated logical reasoning akin to standardised causality assessment. There were 11 (7%) respondents who described fairly rapid onset of the symptom after initial use, 20 (14%) that the symptom began on starting a new medicine and 2 (1%) that they had not experienced it prior to using the suspect medicine. Most gave multiple reasons for suspecting a medicine in relation to timing, for example, *‘Never had this before & it started after I had taken the tabs for a week. Went away when I stopped’* (female, 71–80 years, retired, 5–8 medicines). There were 23 (16%) who described resolution of their symptoms on stopping the medicine (de-challenge).

### Use of information sources and respondents’ confidence levels

Respondents’ confidence that a medicine had caused their symptom was high. Of 158 respondents, 100 (63.3%) were very confident and a further 44 (27.8%) fairly confident, with only 12 (7.6%) judging themselves to be not very confident and 2 (1.3%) not at all confident that a medicine was associated with their symptom.

There was a trend towards increased use of information sources with increasing confidence in the association, although this was not statistically significant. Those who were very confident used an average of 2.56 sources, those fairly confident 2.43 and those not very confident 2.21 sources.

### Consequences of side effects

For many respondents, the adverse effect had an impact on their use of health services: 104 saw a doctor (65.4%) and 14 (8.8%) were admitted to hospital. However, only five judged the side effect to be very serious (3.1%), while 56 (35.0%) considered it serious enough to affect everyday activities, 73 (45.6%) unpleasant, but not affecting daily activities and 26 (16.3%) mild. Of 158 respondents, 19 (12.0%) thought the impact on them was severe, 50 (31.6%) moderate, 67 (42.4%) mild and 22 (13.9%) that it had no impact.

### Impact of side effects and text box responses

In free-text comments, the majority (125; 79.1%) described the physical symptoms they experienced from medicines, while 17 (10.6%) described psychological impacts: *‘… was causing my heart beat to beat faster which made me very anxious and was very unpleasant’* (female, 41–50 years, 1 medicine) and 15 (9.5%) described social impacts: *‘Skin rashes (head & face) caused embarrassment when going out. Dizziness, avoided going out on my own’* (female, 61–70 years, retired, 1 medicine).

There were 92 (57.5%) who stopped taking the medicine because of the side effect; 60 of these were advised to do so by a healthcare professional, 5 by relatives/friends and 27 made the decision themselves.

### SECope and coping behaviours

Factor analysis indicated the revised 10-item scale was reliable – Cronbach’s alpha (*α* = 0.8). The amended SECope contained the same four sub-scales as the original 16-item instrument: information seeking (4 items), non-adherence (2 items), social support seeking (2 items) and taking medicines (2 items). Both the full scale (Cronbach’s *α* = 0.8) and all four sub-scales had acceptable internal reliability: information seeking (*α* = 0.79), non-adherence (*α* = 0.56), social support seeking (*α* = 0.5) and taking medicines (*α* = 0.78).

Responses to individual items showed that over 70% of respondents indicated they would always or often seek information if they experienced a side effect, whereas only 16% indicated they would always or often either reduce the dose or take another medicine to deal with the side effect. The proportion who would stop the medicine was 31%, while the proportion who would continue to take it was similar at 30%. Support-seeking behaviours were anticipated by 40%–45% of respondents (Table 4).

**Table 4.** Responses to SECope (*n* = 230)

Statement	Domain	Never	Rarely	Sometimes	Often	Always
Reduce the dose of the medication that is causing the side effect	Medicine use	103 (46.8%)	35 (15.9%)	49 (22.3%)	22 (10.0%)	11 (5.0%)
Take another medication to deal with the side effect	Medicine use	83 (38.1%)	46 (21.1%)	54 (24.8%)	21 (9.6%)	14 (6.4%)
Decide that the benefit from the medication is not worth the side effect and stop taking it	Adherence	40 (18.2%)	49 (22.3%)	63 (28.6%)	42 (19.1%)	26 (11.8%)
Accept the side effect and take the medication as prescribed	Adherence	55 (25.2%)	33 (15.1%)	64 (29.4%)	34 (15.6%)	32 (14.7%)
Get support from other people	Social support	30 (13.7%)	29 (13.2%)	62 (28.3%)	58 (26.5%)	40 (18.3%)
Talk to family friends loved ones about the problem	Social support	22 (10.0%)	36 (16.3%)	75 (33.9%)	50 (22.6%)	38 (17.2%)
Talk to your doctor or healthcare professional about the problem	Information seeking	6 (2.7%)	13 (5.8%)	42 (18.8%)	59 (26.3%)	104 (46.4%)
Try to get more information about the medication or side effect	Information seeking	7 (3.2%)	18 (8.3%)	36 (16.5%)	64 (29.4%)	93 (42.7%)
Request a medication from your doctor to help with the side effect	Information seeking	52 (23.7%)	38 (17.4%)	67 (30.6%)	31 (14.2%)	31 (14.2%)
Ask your doctor to prescribe a different medication	Information seeking	12 (5.4%)	25 (11.2%)	56 (25.0%)	56 (25.0%)	75 (33.5%)

Respondents who had actually stopped a medicine after experiencing a side effect had significantly higher scores on the non-adherence sub-scale (7.2 ± 2.1) compared to those who did not stop their medicine (4.8 ± 1.8) (*P* < 0.001). Higher scores on the support-seeking scale were associated with predicted use of friends and family as information sources (6.7 ± 1.6 versus 6.02 ± 2.1; *P* = 0.027), but not with actual seeking of information from family and friends (6.4 ± 1.9 versus 6.0 ± 2.1). Respondents who used a health professional as a source of information in relation to a side effect scored more highly on the information-seeking sub-scale (14.6 ± 2.9) than those who did not (11.6 ± 4.9) (*P* < 0.001). However, there was no correlation between scores on this sub-scale and the number of information sources used after experiencing an ADR.

### MBSS and coping styles

All but six respondents completed the MBSS, with the majority categorised as high monitors (165; 73.7%). MBSS status was not related to age, gender, ethnicity, educational status, whether respondents used medicines or experienced a side effect. There was no difference in the mean number of information sources used depending on MBSS score. Although there were no differences in the proportions of high and low monitors who stopped taking their medicine following a side effect, significantly more high monitors took the decision to stop themselves. In addition, high monitors were more likely than others to indicate that sometimes, often or always their responses to a side effect would be to decide that the benefit was not worth the side effect and stop taking it or to ask their doctor to prescribe a different medicine (Table 5).

**Table 5.** Differences in likely actions following an ADR, based on MBSS category

Action/predicted action	*n*	Monitor	Low monitors	*P*-value
Stopped medicine when experienced SE	150	64 (59%)	24 (59%)	0.56
Decided to stop themselves	91	24 (36%)	2 (5%)	0.01
Would sometimes, often or always stop medicine	215	99 (62%)	29 (54%)	0.36
Would sometimes, often or always ask for a different medicine	217	139 (85%)	42 (76%)	0.12

## Discussion

This survey found that the large majority of people would seek information about side effects from GPs and PILs, both of which have been found as preferred information sources previously, both in England and other countries (Hughes *et al.*, 2002; Nähri and Helakorpi, 2007; Munksgaard *et al.*, 2011; Clarke *et al.*, 2016). Our study demonstrated that these sources were also most frequently used after actually experiencing a side effect. In contrast, although two-thirds said they would seek information from a pharmacist, in practice only 28% of those experiencing a side effect had done so. The internet was judged to be a trustworthy source of information by only 15%, but 55% said they would use it and in practice 38% had used it. A systematic review of information-seeking behaviour in relation to health found the internet to be cited in 86% of articles, thus likely to be the most frequently used source of information (Clarke *et al.*, 2016). One reason for such use could be the relatively poor accessibility of doctors, which has been found elsewhere (Clarke *et al.*, 2016). PILs were also used by 67% of respondents, despite fewer considering them to be trustworthy, particularly those using regular medicines. The finding that pharmacists were judged to be both accessible and trustworthy and that 67% claimed they would seek information from a pharmacist could have been related to the survey being distributed in pharmacies. Research elsewhere has shown that pharmacists, along with GPs, are judged as reliable and trusted information sources (Hamrosi *et al.*, 2014). However, a study in Greece found pharmacists to be the least used source of information about medicines (Stavropolou, 2012). Friends and family were infrequently used by our respondents, but studies elsewhere suggest that these may be important sources among people who have no internet access (Clarke *et al.*, 2016).

Our findings suggest that a hierarchy of characteristics may exist in relation to information sources. GPs and PILs were identified as the most commonly used sources with GPs assessed as the most trustworthy and PILs as the most accessible sources, respectively. PILs, which are readily available to medicine users, were actually used by a majority of respondents despite the mixed assessments they received. Respondents’ positive perception of PILs as an accessible information source seemed to mitigate the influence of the other characteristics and resulted in high usage of PILs by respondents. In contrast, GPs were judged to be much less accessible; however, the GPs’ ability to tailor information to the individual, through having all relevant medical information about that individual may be of greater importance than accessibility (Ruppel and Rains, 2012). Individual characteristics such as accessibility, trustworthiness, ease of understanding and relevance could be ranked in importance by patients seeking health information. If accessibility is considered a key characteristic it may explain the frequent use of PILs and the internet. This could also help to explain the lower use of pharmacists, who may have information about an individual’s prescribed medicines, but not someone’s full medical history, with the result that they are less able to tailor information to the individual, despite being seen as accessible.

The confidence which respondents reported in the association between the adverse event they experienced and their medicine showed some relation to the number of information sources they accessed, hence ensuring access to multiple reliable information sources is important. Respondents to previous studies have also expressed high levels of confidence in the association and have described using temporal associations, health professionals and PILs as mechanisms to aid identification (Krska *et al.*, 2011; Chaipichit *et al.*, 2014). In contrast, a qualitative study of patients admitted to hospital as a result of a confirmed ADR found the majority had not attributed their symptoms to a medicine (Lorimer *et al.*, 2012).

Sources such as PILs and the internet, although viewed as trustworthy by fewer people, are probably most likely to be used in addition to seeking information from a health professional (Clarke *et al.*, 2016). The majority of respondents experiencing a side effect had in fact accessed at least one health professional, which is reassuring, as self-diagnosis could, in some cases, lead to inappropriate discontinuation of a medicine. In practice, 58% indicated that they had stopped a medicine because of the side effect they experienced. Doing so on their own initiative was more frequent in those assessed as high monitors, and stopping a medicine was also associated with higher scores for non-adherent coping strategies. However, the majority who had stopped their medicines did so on the advice of a health professional, which is perhaps unsurprising given that a large proportion of the events experienced were serious enough to cause them to see a doctor.

The SECope sub-scales for non-adherence and information seeking showed positive associations with actually stopping a medicine and seeking information from a health professional. This instrument could thus potentially help to predict behaviours in the event of a side effect. However, given the high proportion of respondents categorised as monitors, which is unsurprising as they are more likely than blunters to complete health-related surveys, information seeking is a common finding. Other research has found that patients who seek medicine information from independent sources are more likely to engage in non-adherent behaviours (Carter *et al.*, 2013). In contrast, de Smedt *et al.* (2011), who developed the SECope, found that patients who sought information were likely to take an additional medicine to cope with a side effect. These authors also found that social support seeking was the most common coping strategy in their population, whereas our data show that information seeking was more frequent. Again, this could be related to the large proportion of high monitors in our sample.

## Strengths and Limitations

Our study sample is in line with previous research into pharmacy users which found that women, older people were more likely to collect prescription medicine; and use of pharmacies increases in older age groups but declines in the oldest age group (Bennett and Jones, 2000; Boardman *et al.*, 2005).

This study explored the use of information sources in relation to experiences of side effects from medicines and is the first to do so with reference to coping styles, using a gold standard psychological scale – the MBSS. However, it should be acknowledged that coping styles are not stable across situations and an individual’s coping style can change over time and particularly with situational context. This may restrict the value of the MBSS and may indicate why it did not predict information-seeking strategies following a side effect.

The survey instrument was robust and had high content validity, as it was developed iteratively including two pilots conducted with people known to have used medicines and to have experienced a side effect. This also ensured that the length of the instrument was acceptable, by reducing duplication and optimising question wording and ordering. Although the survey was distributed in community pharmacies, respondents were asked to return completed questionnaires by post or in sealed envelopes to reduce any obsequiousness bias. To overcome selection bias, each customer that was waiting for a prescription was approached. The response rates for the main study were lower than the pilots and to previous surveys using a similar distribution method (Saramunee *et al.*, 2016; Krska *et al.*, 2017). The distribution method, however, prevented the use of reminders that may have improved response rates. In addition, no information could be gathered about non-responders. Hence, the results may reflect a population with specific interest in side effects from medicines, perhaps based on their experiences. In reality, there was no requirement for respondents to have experienced a side effect or be using a regular medicine, as we sought to obtain views on sources of information and hypothetical coping strategies, regardless of actual side effect experience. For those who had experienced a side effect, the survey instrument was structured to facilitate recall of this event. However, recall bias is a potential further limitation.

## Conclusion

Seeking information when experiencing a symptom which could be a side effect from a medicine is a common strategy, potentially predicted using the SECope instrument, but not the MBSS. Doctors, regarded as trustworthy sources of information, are frequently used when such effects occur. However, their limited accessibility could contribute to high use of the internet as an alternative or additional information source. PILs were not considered as trustworthy by many people using regular medicines, but were nonetheless used frequently due to easy accessibility. Further work is needed to identify how these documents, ubiquitous in many countries, can be improved to increase trustworthiness. Reasons for not seeking information from pharmacists, despite their accessibility and trustworthiness, also need investigation.
